# Etiology and Epidemiology of the Typhus Fever of Eastern Europe

**Published:** 1919

**Authors:** 


**Etiology and Epidemiology of the Typhus Fever of Eastern Europe.** By Drs. G. Baehr and H. Plotz. *Journal of the American Medical Association.*

The bacteriologic studies made on sixty-four individuals with the disease demonstrated that the *Bacillus typhi-exanthematici* is regularly present in the blood of people suffering from the typhus fever of eastern Europe. In Volhynia anerobic blood cultures were positive in nineteen out of twenty-four cases, or 79 per cent. of the cases studied. In the Balkans, where blood cultures often could not be placed in an incubator until four or five days after they were taken, the percentage was lower, 47.5. The relative bacteremia was also proportional to the severity of the disease. For example, in the Russian typhus in which the mortality was 5.5 per cent., and in the Balkan 1915-1916 typhus, in which it was 11 per cent., the bacteremia was greater than in the New York series in which the mortality was negligible, 0.2 per cent.; it was much less than in the Mexican typhus (mortality about 20 per cent.) or the severe Balkan type of the disease (mortality from 18 to 60 per cent.). It was found, furthermore, that during chills enormous numbers of organisms may be present in the blood, the maximum being 1,183 colonies in 13 cc. of blood. Morphologically, culturally and serologically, the organisms recovered from these Russian and Balkan patients were identical with those originally recovered in the New York cases. The *Bacillus typhi-exanthematici* has therefore now been recovered by the authors and others from typhus fever patients in the United States, Mexico, Serbia, Bulgaria, Austria and Russia. The maximum titer of agglutinins for *Bacillus typhi-exanthematici* in the blood of the cases studied was 1 : 1,600; the average was 1 : 200. As a result of systematic studies carried out in 100 cases of typhus fever, it was found that agglutinins usually first appear in the blood on about the tenth or eleventh day of the disease, or at about the time when the temperature begins to break. The agglutinin titer of the serum then rapidly increases as the temperature drops to normal, and reaches its maximum toward the end of the first or the beginning of the second week of convalescence. At this time 97 per cent. of the serums were positive. After this time the agglutinins diminish in amount very slowly, and usually persist in the blood for many months. The curve of the development of specific agglutinins in typhus fever is therefore a typical immunity curve. During the course of the work, specific agglutinins and complement-fixing antibodies were found in the blood of twenty normal persons, in amounts otherwise observed only in convalescents from typhus fever. None of these persons had ever shown any clinical manifestations of typhus fever. These twenty patients were either physicians, nurses or male attendants in typhus hospitals in which they were constantly handling typhus patients, or they were peasants who had been living with relatives or friends ill with the disease. As these twenty contacts were the only persons, aside from typhus convalescents, in whom such findings were made, it was reasonable to assume that during the time of their exposure to typhus fever they had actually been infected with the bacilli in quantities sufficient to induce the clinical manifestations of the disease, and had reacted with the production of specific antibodies. Such persons, who have probably become immune without having the disease, may act as carriers of infected lice and be agents of their distribution throughout a community.

